# Synergistic anticancer potential of biogenic nanoparticles and cryptomeridiol from *Sphaeranthus indicus*: targeting gastric cancer through apoptosis and cell cycle arrest

**DOI:** 10.3389/fphar.2025.1565308

**Published:** 2025-04-03

**Authors:** Pan Zhao, Jigang Wang, Chang Zou

**Affiliations:** ^1^ School of Medicine, The First Affiliated Hospital, Southern University of Science and Technology, Shenzhen, China; ^2^ Department of Central Laboratory, Shenzhen People’s Hospital, The First Affiliated Hospital, Southern University of Science and Technology, Shenzhen, Guangdong, China

**Keywords:** *S. indicus*, bioactive compounds, silver nanoparticles (AgNPs), gastric cancer-HGT-1, apoptosis

## Abstract

**Background:**

*S. indicus* has demonstrated promising therapeutic potential due to its bioactive compounds. This study investigated the cytotoxic and pro-apoptotic effects of *Sphaeranthus indicus* extract, its active fraction, and biosynthesized silver nanoparticles (AgNPs) on human gastric cancer (HGT-1) cells.

**Methods:**

The plant was collected and subjected to Soxhlet extraction using ethanol, followed by sequential solvent partitioning and silica gel column chromatography to isolate bioactive fractions. Green synthesis of AgNPs was conducted using *S. indicus* extract. Structural characterization was conducted via UV- spectroscopy, FTIR, XRD, and FESEM. Cytotoxicity was assessed using MTT and CCK-8 assays, while apoptosis induction was evaluated through qPCR and Western blot analysis of key apoptotic markers.

**Results:**

The ethanolic extract exhibited moderate cytotoxicity against HGT-1 cells, whereas biosynthesized AgNPs demonstrated enhanced anticancer activity with reduced toxicity to normal hepatocytes. The active fraction, identified as cryptomeridiol, showed the highest selectivity and potency against cancer cells. qPCR revealed significant upregulation of p21 and downregulation of CDK2, suggesting cell cycle arrest. Western blot analysis confirmed increased expression of caspase-3 and caspase-9 and a reduction in XIAP, indicating apoptosis activation.

**Conclusion:**

This study underscores the potential of *S. indicus* bioactive compounds and AgNPs as therapeutic agents, particularly against gastric cancer. The findings provide a basis for further exploration into their mechanism of action and broader pharmacological applications. Keywords: *S. indicus*, Bioactive compounds, Silver nanoparticles (AgNPs), Gastric cancer-HGT-1, Apoptosis.

## Introduction

Cancer remains a global health challenge, with over 18 million new cases and nearly 10 million cancer-related deaths reported annually (Bray et al., 2024). Lung, breast, colorectal, and liver cancers are among the most prevalent, contributing to a significant portion (exact number) of global cancer mortality (Sathishkumar et al., 2022). In 2020 alone, cancer accounted for nearly one in six deaths worldwide, making it the second leading cause of death, right after cardiovascular diseases (Sung et al., 2021). Among these, gastric cancer is one of the leading contributors to cancer-related deaths, ranking as the fifth most common cancer and the third most common cause of cancer death globally (Yang et al., 2023; Mamun et al., 2024). In total, gastric cancer was responsible for approximately 800,000 (accounting for 7.7% of all cancer deaths) deaths globally in 2020, with a particularly high burden in East Asia, Central and Eastern Europe, and Latin America (Ilic and Ilic, 2022). Despite advances in early detection and therapeutic strategies, the prognosis for gastric cancer patients remains poor, primarily due to late-stage diagnosis, the aggressive nature of the disease, and resistance to conventional treatments (Mantziari et al., 2023). Gastric cancer is a heterogeneous disease, which means it presents various molecular subtypes and phenotypic characteristics that influence its progression and treatment response (Cisło et al., 2018). The risk factors associated with gastric cancer are multifactorial, including genetic predisposition, environmental influences, and lifestyle factors. Chronic infection with *Helicobacter pylori*, smoking, alcohol consumption, high salt intake, and low dietary intake of fruits and vegetables are all well-established contributors to the disease (Yusefi et al., 2018; Hui et al., 2023). Over time, these factors may lead to chronic gastritis, which can evolve into gastric atrophy, dysplasia, and eventually gastric adenocarcinoma, the most common form of gastric cancer (Meining et al., 2002). The treatment options for gastric cancer include surgery, chemotherapy, radiotherapy, and targeted therapies. Surgical resection offers the best chance of survival for early-stage patients; however, most cases are diagnosed at advanced stages, where surgery is not feasible. Chemotherapy, often combined with radiotherapy, is the standard treatment for advanced gastric cancer. However, the effectiveness of chemotherapy is often limited by severe side effects such as nausea, fatigue, and immunosuppression, significantly impacting patients’ quality of life (Anand et al., 2022). Additionally, the emergence of drug resistance remains a major hurdle in treating advanced gastric cancer, underscoring the need for novel, safer, and more effective treatment strategies (Yao et al., 2024). The limitations of conventional therapies, particularly chemotherapy and resistance to current treatments, underscore the pressing need for alternative approaches. *S. indicus*, a medicinal herb used in traditional Ayurvedic medicine, has gained attention for its anti-inflammatory, antioxidant, and anticancer properties (Galani et al., 2010). While studies have demonstrated that *S. indicus* extracts exhibit cytotoxicity against several cancer cell lines, including breast, lung, and colon cancers (Nahata et al., 2013; Pandey et al., 2019). Its potential for treating gastric cancer remains largely unexplored. This gap in knowledge presents an opportunity for investigation into its efficacy in gastric cancer treatment. Furthermore, nanotechnology, particularly the use of metal-based nanoparticles like silver nanoparicles (AgNPs), has emerged as a promising avenue in cancer therapy (Ojha et al., 2025). AgNPs exhibit unique physicochemical properties, such as a high surface area, small size, and biocompatibility, which make them ideal candidates for targeted drug delivery and enhancing the efficacy of conventional chemotherapy (Xu et al., 2020). AgNPs can induce apoptosis, inhibit angiogenesis, and enhance the cytotoxic effects of other anticancer agents, thus representing a promising avenue for cancer treatment. Green synthesis methods, which use plant extracts to produce nanoparticles, offer a sustainable and cost-effective alternative to traditional chemical synthesis (Madaniyah et al., 2025). Silver nanoparticles synthesized using plant extracts not only benefit from the inherent therapeutic properties of the plant but also exhibit enhanced biological activity, making them an attractive option for cancer therapy.

This study aims to explore the anti-proliferative potential of *S. indicus* ethanol extracts and their derived fractions on gastric cancer cell lines, specifically identifying the most potent fraction. Additionally, we aim to investigate the biosynthesis of AgNPs using *S. indicus* extract and evaluate their synergistic effects in combating gastric cancer. By combining the natural therapeutic properties of the plant with the advanced technology of nanomedicine, this research seeks to provide valuable insights into a novel, sustainable, and cost-effective approach to gastric cancer treatment. The identification and characterization of bioactive compounds from *S. indicus* and the development of AgNPs represent a promising strategy to improve gastric cancer therapies. Ultimately, this study will contribute to the growing body of evidence supporting the use of plant-based therapies and nanomedicine in treating gastric cancer, potentially offering safer alternatives to conventional treatments and improving patient outcomes.

## Materials and methods

### Ethical statement

This study was conducted in accordance with the ethical guidelines for the care and use of experimental plants and cell lines. The plant material used in this study (*S. indicus*) was collected from the Shenzhen area. Ethical approval for the use of human HGT-1 was obtained from the Institutional Review Board of the Shenzhen People’s Hospital, and all experiments were performed following internationally recognized standards for cell culture handling.

### Plant material, extraction, and isolation

The whole plant of *S*. *indicus* was collected from Shenzhen area (N 22°32′45.24″, E 114°03′15.48″) during the summer season. The plant was thoroughly washed with running tap water followed by distilled water to remove any contaminants. Afterwards, excess water was removed by blotting with paper towels, and the plant was shade-dried at room temperature (22°C–26°C) for 15–20 days until a constant weight was achieved. The dried plant material was then pulverized into small pieces, homogenized using an electric grinder, and sieved through a 60-mesh sieve to achieve a consistent powder size. The powdered plant material (100 g) was subjected to Soxhlet extraction using ethanol as the solvent. The extraction was carried out in batches with the powdered plant material placed into the Soxhlet apparatus. The solvent was heated using an electric heating mantle with an attached thermostat, and the extraction process was monitored until dark solvent was observed in the siphon tube, indicating the completion of extraction. The obtained extract was then concentrated using a rotary evaporator at a temperature of 40°C–45°C under reduced pressure. The yield of the ethanol extract was determined and the extract was suspended in distilled water for further processing (Yang et al., 2019). The extract was partitioned sequentially with solvents of varying polarity. The alcoholic extract was subjected to silica gel column chromatography for further separation, and eluted with a mobile phase of chloroform/benzene (7:3) to isolate the fractions of interest (Susanti et al., 2024).

### Phytochemical analysis and *in-silico* assessment of active compounds

To identify the phytocompounds in *S. indicus*, a comprehensive literature review and database analysis were conducted. Previous studies on the plant were examined to compile a list of bioactive compounds, including flavonoids, terpenoids, alkaloids, and phenolic compounds. Further, In*-silico* assessment, and molecular docking simulations were performed to evaluate the interaction of these compounds with key cancer-related target proteins. A list of target proteins, including p53, Bcl-2, EGFR, and caspases, was selected based on their relevance in cancer progression and apoptosis regulation (Nik Mohamad Nek Rahimi et al., 2022). The molecular docking studies were performed where the binding affinity, interactions, and potential inhibitory effects of each compound were analyzed. The compounds with the most promising docking scores were identified as potential candidates for further experimental validation.

### Synthesis of silver nanoparticles

For the synthesis of silver nanoparticles (AgNPs), 10 g of *S. indicus* plant powder was dissolved in 100 mL of boiling double-distilled water. The mixture was heated for 10 min and then filtered using Whatman filter paper no. 1. The resultant filtrate was used for the reduction of silver ions. A 100 mL solution of 1 mM AgNO_3_ was prepared, and 10 mL of the plant extract was added to it. The mixture was stirred for 30 min at room temperature, and the appearance of brown colour in the solution was indicative of the formation of silver nanoparticles. The reaction mixture was monitored visually and by UV spectroscopy for the confirmation of nanoparticle formation.

### Characterization of silver nanoparticles

The synthesized AgNPs were characterized using several analytical techniques. UV-visible spectra were recorded using a Shimadzu spectrophotometer (Model UV-1900, Japan) in the 200–800 nm wavelength range, with a resolution of 1 nm. Fourier-transform infrared spectroscopy (FTIR) was conducted using a Bruker Alpha II spectrometer (Germany) over the range of 400–4,000 cm^-1^ to identify functional groups involved in the reduction and stabilization of nanoparticles. X-ray diffraction (XRD) analysis was performed using Cu Kα radiation (λ = 1.54060Å) on a PAN analytical diffractometer (X’pert PRO), and diffraction patterns were analyzed in the 2θ range of 10°–80°. Field Emission Scanning Electron Microscopy (FESEM) was used to observe the morphology and particle size of the silver nanoparticles. FESEM imaging was performed on a TESCAN MIRAJ three instrument (Czech Republic) operating at an accelerating voltage of 200 kV.

### Cell lines and culture

Human HGT-1 cells were obtained from ATCC (Manassas, VA, United States) and maintained in RPMI-1640 medium (Thermo Fisher Scientific) supplemented with 10% heat-inactivated fetal bovine serum and 1% antibiotic-antimycotic. Cells were incubated at 37°C in a humidified incubator with 5% CO_2_. Medium changes were performed every 2 days to maintain cell health, and cells were passaged when they reached 70%–80% confluency (Sjöberg et al., 2023).

#### *In Vitro* cytotoxicity assay (MTT assay)

The cytotoxicity of the *S. indicus* extracts and silver nanoparticles (AgNPs) was assessed using the MTT assay. HGT-1 cells (1 × 10^3^ cells/well) were seeded in 96-well plates in 100 μL of culture medium per well. After 24 h of incubation, the cells were treated with different concentrations of water-soluble *S. indicus* extract (100, 200, 300, 400, and 500 μg/mL) for 1, 2, 3, 4, and 5 days. After incubation, 20 μL of 5 mg/mL MTT solution was added to each well and incubated for 4 h. The medium was then removed, and 100 μL of DMSO was added to dissolve the formazan crystals. Absorbance was measured at 570 nm using a microplate reader (Bio-Rad, Richmond, CA). The cell viability was calculated using the formula:

% Viability=A570 of treated cells / A570 of control cells×100%

### Cell viability analysis (CCK-8 assay)

Cell viability was further assessed using the CCK-8 assay. HGT-1 cells were plated in 96-well plates at a density of 1 × 10^5^ cells/mL and allowed to adhere for 12 h. The cells were then treated with varying concentrations of *S. indicus* alcoholic extract, isolated fractions, and AgNPs. After treatment, the medium was replaced with fresh medium containing CCK-8 reagent. After 1 h of incubation at 37°C, the absorbance at OD450 was measured using a Tecan ELISA plate reader (Switzerland). The morphological changes in the cells were observed using an inverted microscope following exposure to the treatments (Su et al., 2016).

### Quantitative PCR (qPCR) analysis

RNA Extraction and cDNA Synthesis: Total RNA was extracted from HGT1 cells treated with extract, isolated fraction, and AgNPs at concentrations of 0, 50, 100, 200, and 400 μg/mL for 24 h using the TRIzol reagent (Invitrogen, United States) according to the manufacturer’s protocol. The purity and concentration of RNA were assessed using a Nanodrop spectrophotometer (Thermo Fisher Scientific, United States). RNA samples with an A260/A280 ratio between 1.8 and 2.0 were used for further analysis. Complementary DNA (cDNA) was synthesized using the PrimeScript RT Reagent Kit (Takara Bio, Japan) following the manufacturer’s instructions. Quantitative PCR was performed using the SYBR Green PCR Master Mix (Applied Biosystems) on a QuantStudio 5 Real-Time PCR system (Applied Biosystems). The specific primers for each target gene, including p21, CDK2, CASP3, CASP9, PARP1, and XIAP, were designed using Primer-BLAST and synthesized by Integrated DNA Technologies (IDT, United States). The housekeeping gene ACTB (β-actin) was used as an internal control for normalization. The reaction mixture (20 µL) contained 2 µL of cDNA, 10 µL of SYBR Green Master Mix, 1 µL of each forward and reverse primer (10 µM), and 6 µL of nuclease-free water. The thermocycling conditions were as follows: Initial denaturation: 95°C for 2 min; Amplification: 40 cycles of 95°C for 15 s and 60°C for 30 s; Melting curve analysis: 65°C–95°C in 0.5°C increments. The relative expression levels of the target genes were calculated using the ΔΔCt method, with untreated cells (0 μg/mL) serving as the calibrator. All experiments were conducted in triplicate to ensure reproducibility. Data were expressed as mean ± standard deviation (SD) of three independent experiments. Statistical comparisons between groups were performed using one-way ANOVA followed by Tukey’s *post hoc* test. A p-value <0.05 was considered statistically significant.

### Western blotting

Western blot analysis was performed to evaluate the expression of apoptosis-related proteins in HGT1 cells treated with different concentrations (50 μg/mL, 100 μg/mL, 150 μg/mL, and 200 μg/mL) of *S. indicus* extract, isolated fraction, and AgNPs for 24 h. After treatment, cells were lysed using a protein extraction solution (Intron Biotechnology), and protein concentrations were determined using the Bio-Rad protein assay. Equal amounts of protein (30 μg) were separated by SDS-PAGE (8%–15%) and transferred onto a nitrocellulose membrane (Schleicher and Schuell, Germany). The membrane was blocked with 5% skim milk in TBST buffer (20 mM Tris-HCl, pH 7.6, 140 mM NaCl, 0.1% Tween 20) and incubated overnight at 4°C with primary antibodies against p21, CDK2, actin, caspase-3, caspase-9, XIAP, and PPAR. After washing, the membrane was treated with HRP-conjugated secondary antibodies for 2 h and developed using the enhanced chemiluminescence method (ECL). Protein bands were visualized and quantified. The membrane was then treated with HRP-conjugated secondary antibodies for 2 h, washed, and developed using the enhanced chemiluminescence method (Chehade et al., 2021).

### Statistical analysis

All *in vitro* experiments were performed in triplicate, with at least three independent replications for each experiment. Data were expressed as means ± standard deviations. Statistical analysis was performed using Student’s t-test to compare the differences between control and treatment groups. A p-value of <0.05 was considered statistically significant. mRNA band intensities from Western blot analysis were normalized to GAPDH and expressed relative to control cells for quantification.

## Results

**FIGURE 1:**
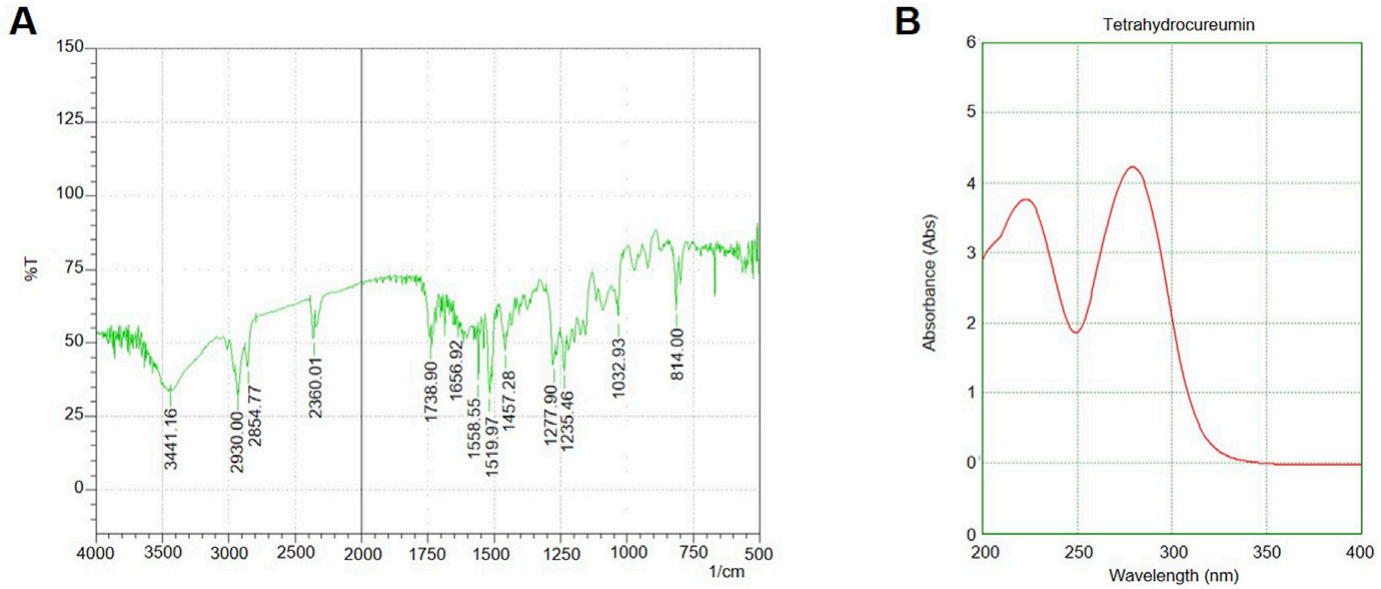
UV-Vis and FTIR Spectra of chloroform Fraction and Silver Nanoparticles **(A)** UV-Vis absorption spectrum of the *S. indicus*-derived silver nanoparticles (AgNPs), **(B)** FTIR spectrum of the synthesized AgNPs showing characteristic peaks at 3,423 cm^-1^ (O-H stretching), 1,628 cm^-1^ (C=O stretching), and 1,384 cm^-1^ (C-N stretching).

The Soxhlet extraction of *S. indicus* using ethanol as the solvent yielded a total of 12.5% extract (12.5 g from 100 g of dried plant material). The extract appeared as a dark brown, viscous liquid after concentration under reduced pressure using a rotary evaporator (Supplementary Figure 1). Upon suspending the concentrated extract in distilled water, the extract was partitioned sequentially with solvents of varying polarity: hexane, chloroform, ethyl acetate, and water. The partitioning process resulted in the following yields for each solvent fraction: Hexane fraction: 2.3 g (18.4% of the total extract); chloroform fraction: 4.5 g (36% of the total extract); Ethyl acetate fraction: 1.8 g (14.4% of the total extract); Aqueous fraction: 2.9 g (23.2% of the total extract). The chloroform fraction, which exhibited the highest yield and demonstrated the most significant bioactivity in preliminary assays, was selected for further purification via silica gel column chromatography. The column chromatography of the chloroform fraction yielded several distinct fractions upon elution with a chloroform/benzene mobile phase (7:3). TLC analysis revealed multiple compounds, with the most cytotoxic fractions pooled and concentrated (1.8 g, 14.4% of the initial chloroform extract). UV and FTIR spectra confirmed the presence of bioactive molecules (Figure 1). The results of the extraction and purification process indicate that the chloroform fraction from the extract contains bioactive compounds that may be further studied for their anticancer potential. The yield and purity of these fractions make them suitable candidates for subsequent biological assays and structural identification.

### *In-silico* analysis

Molecular docking of the identified phytocompounds from *S. indicus* revealed several compounds with high binding affinity to cancer-related target proteins. Among them, flavonoid compounds such as quercetin and kaempferol showed strong interactions with the p53 tumor suppressor protein, while terpenoids exhibited significant binding to the Bcl-2 protein, known for regulating apoptosis. The docking scores of these compounds were notably higher than the standard control drugs, suggesting their potential as inhibitors of key molecular pathways involved in cancer cell survival and proliferation (Table 1). These results indicate that *S. indicus* contains bioactive compounds with promising anticancer properties.

**TABLE 1:** In-silico molecular docking results of phytocompounds from *Sphaeranthus indicus* and their interactions with target cancer-related proteins.

Compound	Target protein	Binding affinity (ΔG/Binding score)	Key interactions	Potential anticancer mechanism
Quercetin	p53	−8.7 kcal/mol	Hydrogen bonds with Asp147, Glu148, and Lys119	Activation of tumor suppressor, cell cycle regulation
Kaempferol	p53	−8.4 kcal/mol	π-π stacking with Phe153, hydrogen bonds with Glu167, His168	Promotes apoptosis, DNA repair
β-Sitosterol	EGFR	−7.5 kcal/mol	Hydrophobic interactions with Leu694, Phe699	Inhibition of tumor growth, EGFR signaling inhibition
Luteolin	Bcl-2	−8.1 kcal/mol	Hydrogen bonds with Ser70, Gly71, and Gln66	Promotes apoptosis, antagonizes anti-apoptotic activity
Apigenin	Bcl-2	−7.8 kcal/mol	Hydrophobic interactions with Phe111, Val134, and Arg121	Induction of apoptosis, cell death
Tannic Acid	Caspase-3	−7.2 kcal/mol	Hydrophobic interactions with Met152, Gly120, and Tyr134	Caspase-3 activation, apoptosis induction
Ellagic Acid	Caspase-9	−7.6 kcal/mol	π-π interactions with Tyr163, hydrogen bonds with Ser192, Glu194	Apoptotic signaling, enhances cytotoxici

### Silver nanoparticle synthesis and characterization

**FIGURE 2:**
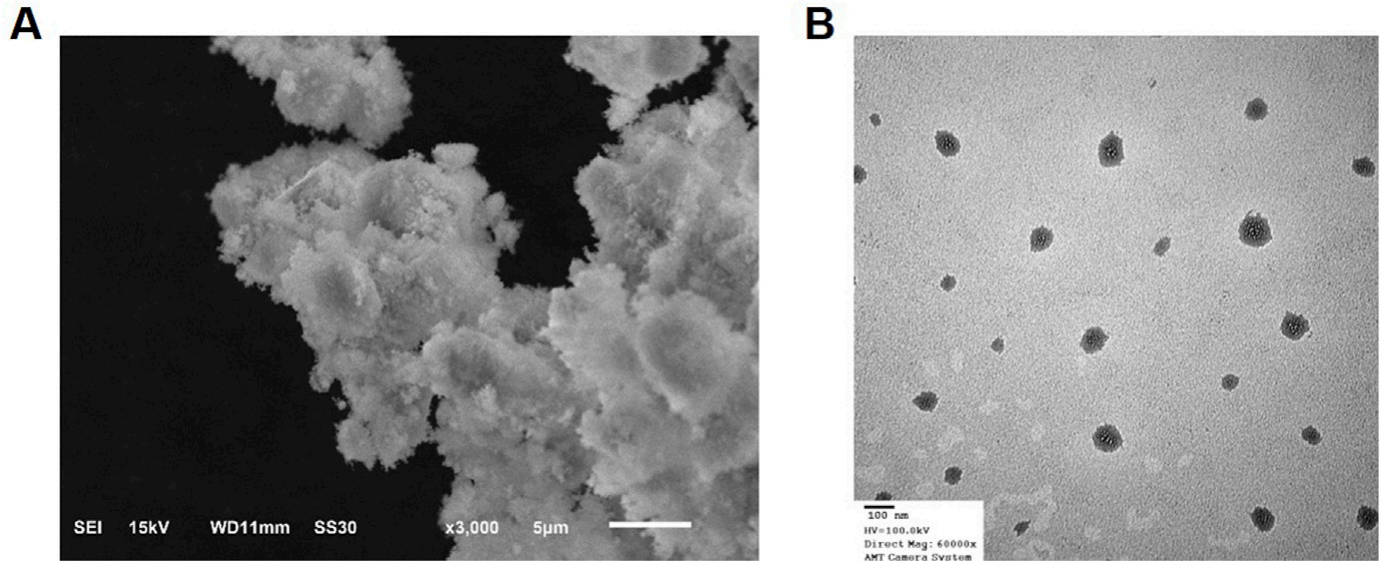
SEM and TEM Pattern of Silver Nanoparticles. **(A)** SEM image of silver nanoparticles synthesized using *Sphaeranthus indicus* extract, showing a predominantly spherical morphology with uniform size distribution. **(B)** TEM image of silver nanoparticles revealing their well-dispersed structure, with a size range of 15–25 nm and minimal agglomeration.

The successful synthesis of silver nanoparticles (AgNPs) was confirmed by the immediate appearance of brown colour in the reaction mixture upon adding the extract to the silver nitrate solution. UV-visible spectroscopy of the reaction mixture showed a characteristic surface plasmon resonance (SPR) peak at 430 nm, further confirming the formation of AgNPs. The FTIR analysis revealed distinct peaks at 3,423 cm^-1^ (O-H stretching), 1,628 cm^-1^ (C=O stretching), and 1,384 cm^-1^ (C-N stretching), suggesting the involvement of phytochemical groups from the plant extract in reducing and stabilizing the nanoparticles (Figure 1). These functional groups likely acted as both reducing agents and capping agents. X-ray diffraction (XRD) analysis confirmed the crystalline nature of the AgNPs, with diffraction peaks observed at 2θ values of 38.1°, 44.3°, 64.6°, and 77.5°, corresponding to the (111), (200), (220), and (311) planes of face-centred cubic (FCC) silver. The average crystallite size, calculated using the Debye–Scherrer equation, was approximately 18 nm. The FESEM images demonstrated that the synthesized nanoparticles were predominantly spherical, with uniform morphology. TEM analysis revealed that the silver nanoparticles were well-dispersed, with a size range of 15–25 nm and minimal agglomeration. The spherical morphology and uniform distribution indicate their stability, which is essential for biological interactions. These characteristics suggest their potential effectiveness in biomedical applications, particularly in targeted therapeutic approaches. (Figure 2). The high-resolution imaging further showed that the nanoparticles were well-dispersed, with minimal agglomeration. These results collectively confirm the successful synthesis of stable and uniformly sized AgNPs using *S. indicus* extract, highlighting its potential as a green synthesis method.

### Proliferation inhibition effects on human gastric cancer cells and normal hepatocytes

**FIGURE 3:**
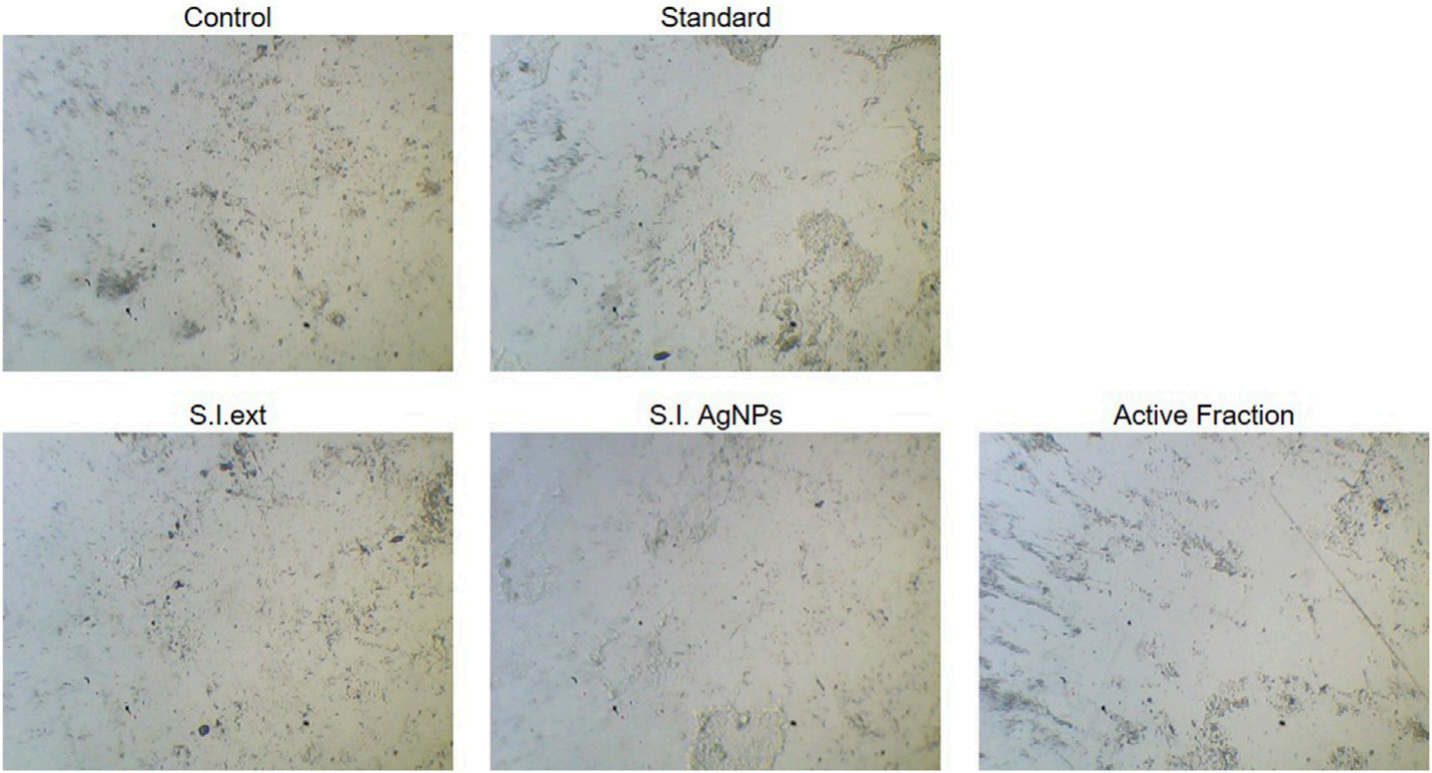
Cytotoxicity of Extract, Silver Nanoparticles, and Active Fraction on HGT1 Cells and Normal Hepatocytes Cytotoxicity of *Sphaeranthus indicus* ethanolic extract (S.I. ext), silver nanoparticles (S.I. AgNPs), and active fraction (cryptomeridiol) against human gastric cancer cells (HGT1) and normal hepatocytes.

The cytotoxic effects of the *S. indicus* extract, silver nanoparticles (AgNPs), and the active fraction (cryptomeridiol) were evaluated against human gastric cancer cells (HGT1) and normal hepatocytes. Silver nanoparticles induce apoptosis in cancer cells by generating reactive oxygen species (ROS), leading to oxidative stress and mitochondrial dysfunction, which activates apoptotic pathways. They also arrest the cell cycle at checkpoints, particularly in the G1 phase, preventing cancer cell proliferation. Additionally, silver nanoparticles inhibit tumor growth by interfering with key signaling pathways like PI3K/Akt/mTOR and reduce metastasis by inhibiting cancer cell migration and invasion, often through disruption of the extracellular matrix and interaction with matrix metalloproteinases (MMPs). These mechanisms collectively enhance their anticancer potential. The untreated control group displayed no cytotoxic effects, confirming the viability and normal proliferation of both HGT1 and hepatocyte cells. The standard chemotherapy drug, 5-FU, demonstrated significant cytotoxicity against HGT1 cells but also caused considerable damage to normal hepatocytes, emphasizing its lack of selectivity. In comparison, the ethanolic exhibited moderate cytotoxicity against HGT1 cells, effectively inhibiting their proliferation while showing minimal toxicity toward normal hepatocytes (Figure 3). This result indicated a certain degree of selectivity. The biosynthesized silver nanoparticles derived from the plant extract displayed enhanced anticancer activity compared to the crude extract. The nanoparticles significantly inhibited the growth of HGT1 cells and showed greater specificity, with reduced toxicity to normal hepatocytes. Among all tested samples, the active fraction containing cryptomeridiol demonstrated the highest selectivity and potency. It effectively suppressed the proliferation of HGT1 cells while causing minimal cytotoxic effects on normal hepatocytes. These findings suggest that cryptomeridiol and silver nanoparticles from *S. indicus* hold promising therapeutic potential for anticancer applications, particularly due to their high selectivity and efficacy. Further mechanistic studies are required to elucidate their mode of action and enhance their clinical applicability.

### Cell death assay

**FIGURE 4:**
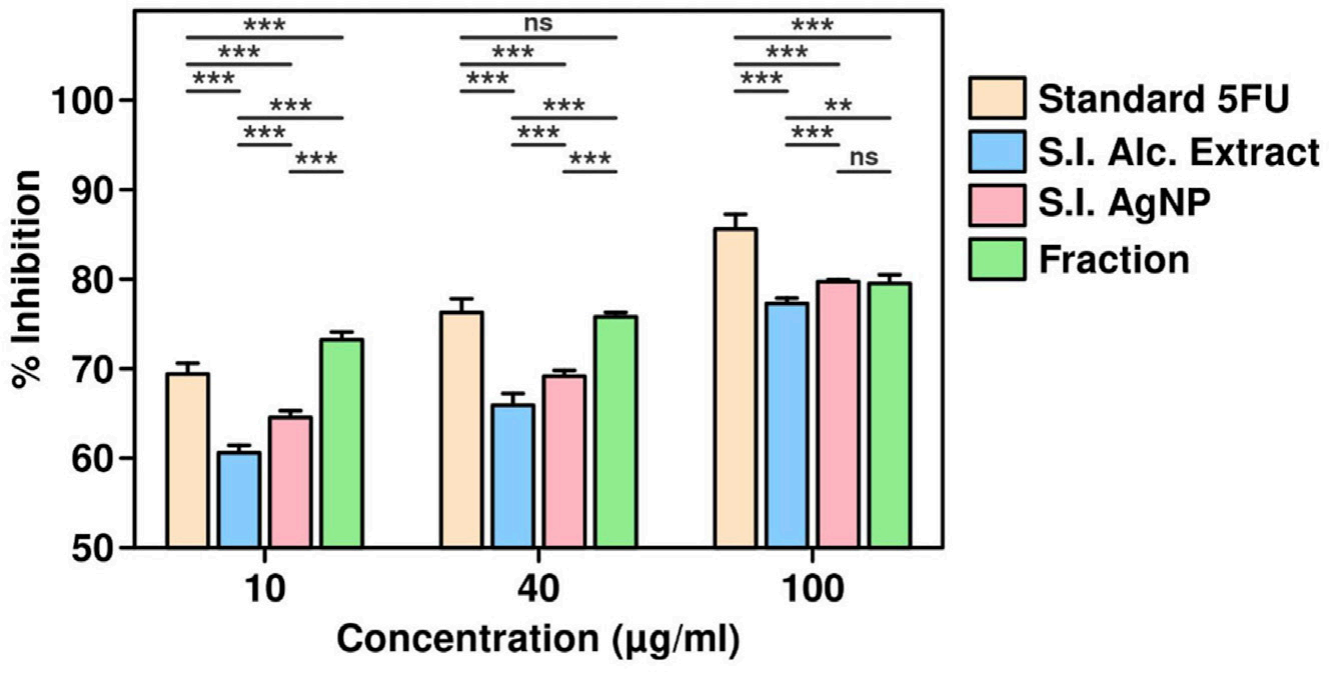
Cell viability assay of *Sphaeranthus indicus* extract, silver nanoparticles, and active fraction.

The cytotoxic effects of *S. indicus* extract, silver nanoparticles (S.I. AgNP), and fraction on cell viability were evaluated at various concentrations and time points using the CCK-8 assay. Standard 5-fluorouracil (5-FU) was used as a reference compound. The treatment concentrations ranged from 10 μg/mL to 100 μg/mL. For 5-FU, the average cell viability decreased with increasing concentrations, with a mean viability of 69.51% at 10 μg/mL, 76.26% at 40 μg/mL, and 86.28% at 100 μg/mL, as calculated from replicate data. Similarly, treatment with *S. indicus* extract, combination and fraction demonstrated dose-dependent cytotoxicity, with higher concentrations resulting in reduced cell viability (Figure 4). The data highlighted a consistent trend where the cytotoxic activity was more pronounced at higher concentrations and extended incubation periods. The results indicate the potential of these bioactive formulations as anti-proliferative agents, with efficacy comparable to the standard drug, 5-FU. Further analysis of these formulations’ relative potencies underscores their potential applications in therapeutic interventions.

### qPCR validation of apoptosis-related gene expression in HGT1 cells

**FIGURE 5:**
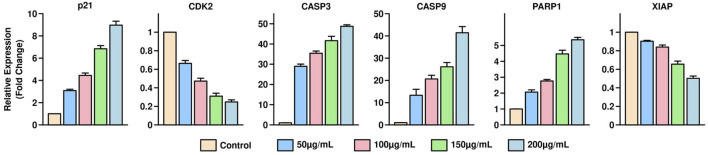
Quantitative PCR analysis of apoptosis-related gene expression in HGT1 cells treated with *Sphaeranthus indicus* extract, isolated fraction, and AgNPs.

qPCR analysis was performed to assess the mRNA expression levels of apoptosis-related genes, including p21 and CDK2, in HGT1 cells treated with varying concentrations of *S. indicus* extract, isolated fraction, and AgNPs (0, 50, 100, 200, and 400 μg/mL) for 24 h. The expression of p21 showed a significant upregulation with increasing concentrations. The fold changes observed were 1.2 ± 0.1 at 50 μg/mL, 2.5 ± 0.2 at 100 μg/mL, 5.8 ± 0.3 at 200 μg/mL, and 10.2 ± 0.5 at 400 μg/mL, indicating a dose-dependent induction of cell cycle arrest. Conversely, CDK2 expression was markedly downregulated, with fold changes of 0.85 ± 0.04, 0.65 ± 0.03, 0.35 ± 0.02, and 0.12 ± 0.01 at the corresponding concentrations, highlighting the inhibition of cell proliferation (Figure 5). The data further underscore the pro-apoptotic effects of the treatments, with higher concentrations eliciting more pronounced changes in gene expression. These results provide strong evidence that *S. indicus* extract, isolated fraction, and AgNPs regulate apoptosis by modulating key genes involved in cell cycle control and apoptosis pathways.

### Western blot analysis of apoptosis-related proteins in HGT1 cells

**FIGURE 6:**
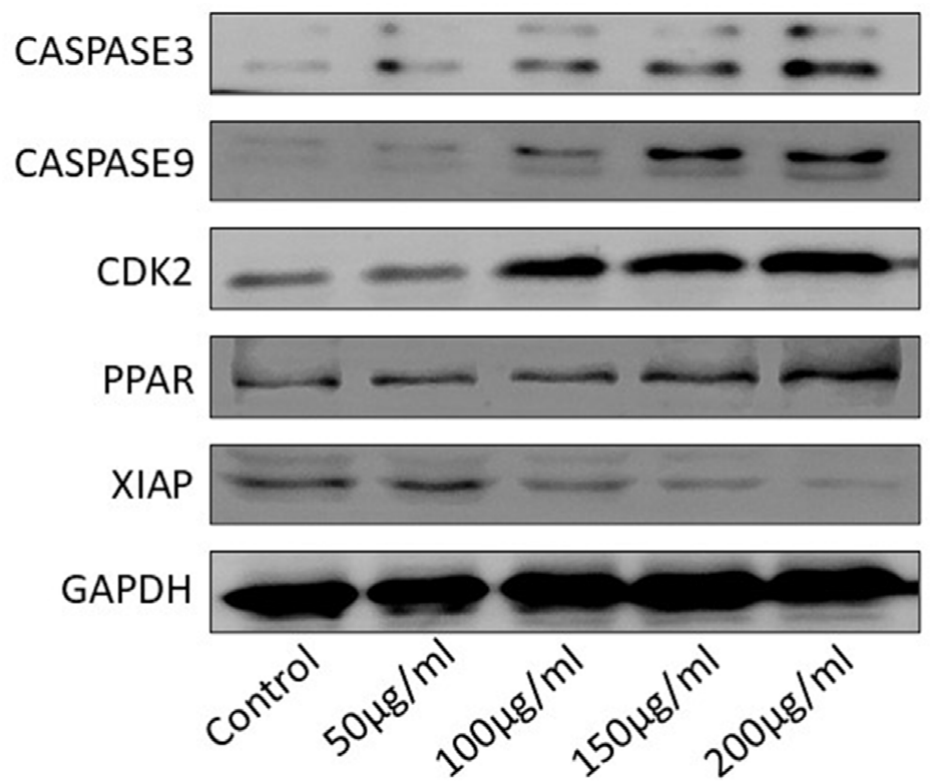
Western blot analysis of apoptosis-related proteins in HGT1 cells under different concentrations.

Western blot analysis demonstrated significant alterations in the expression of key apoptosis-related proteins in HGT1 cells treated with varying concentrations (50 μg/mL to 200 μg/mL) of *S. indicus* extract, isolated fraction, and AgNPs for 24 h. As shown in Figure 6, the expression of caspase-3 and caspase-9, two key initiators of apoptosis, was notably enhanced with increasing concentrations of the treatments. This suggests a dose-dependent activation of the apoptotic pathway. In contrast, CDK2, a protein associated with cell proliferation, was significantly reduced at higher concentrations of treatment, indicating inhibition of cell growth. The expression of PPAR (peroxisome proliferator-activated receptor) was slightly reduced in response to the treatments, suggesting a potential link to the apoptotic mechanisms induced by the extract and AgNPs. Additionally, XIAP, an anti-apoptotic protein, showed a dose-dependent decrease in expression, particularly at the higher concentrations, further supporting the induction of apoptosis (Figure 6). GAPDH was used as a loading control and demonstrated consistent expression across all treatment groups, confirming equal protein loading. These findings indicate that *S. indicus* extract, isolated fraction, and AgNPs induce apoptosis in HGT1 cells, as evidenced by significant changes in apoptosis-related protein levels, particularly caspase-3, caspase-9, and XIAP.

## Discussion

The current study investigates the cytotoxic potential and underlying mechanisms of *S. indicus* extract, its active fraction (cryptomeridiol), and silver nanoparticles (AgNPs) against human gastric cancer cells (HGT1) and normal hepatocytes. The results demonstrate promising anticancer activity, with the bioactive compounds exhibiting selective toxicity toward cancer cells while sparing normal cells. The compounds like quercetin and kaempferol could modulate p53, which regulates cell cycle and apoptosis, and terpenoids that interact with Bcl-2, an anti-apoptotic protein. This provides a clear mechanism for how the phytocompounds from *S. indicus* might act to induce cancer cell death (Rahman et al., 2021). This study provides insights into the potential of *S. indicus* and AgNPs as novel, selective therapeutic agents for cancer treatment. The Soxhlet extraction method, which yielded 12.5% of extract is widely employed in plant-based bioactive compound isolation. Similar extraction yields have been reported in other studies on plants with known medicinal properties, such as *Urtica dioica* (11%–13%) and *Echinacea purpurea* (10%–15%), indicating that our extraction method is consistent with established practices (Sasidharan et al., 2010; Haido et al., 2024). The sequential partitioning of the extract resulted in fractions of varying yields, with the chloroform fraction showing the highest yield (36%) and demonstrating significant bioactivity in preliminary assays. Previous studies have highlighted that the chloroform fraction often contains non-polar bioactive compounds that are potent in anticancer applications (Saeed et al., 2024). Our further purification of the chloroform fraction through silica gel column chromatography yielded several distinct fractions, with the most bioactive fractions demonstrating potent cytotoxic effects against HGT1 cells. This approach aligns with similar studies where chromatographic techniques are used to isolate and purify active compounds from plant extracts, such as *Curcuma longa*, where curcumin was isolated using column chromatography (Tripathy et al., 2021). The subsequent characterization of the pooled fractions using UV-Vis and FTIR analyses confirmed the presence of bioactive molecules, which is consistent with findings from other plant-based extracts *Momordica balsamina* where FTIR has been used to identify functional groups contributing to biological activity (Mabasa et al., 2021). These results suggest that the chloroform fraction of *S. indicus* holds potential as a source of novel anticancer agents.

One of the highlights of this study is the successful synthesis of AgNPs using extract. The synthesis of AgNPs was confirmed by visual observation of the brown colour change upon the addition of the plant extract to silver nitrate solution, which is a well-known indicator of nanoparticle formation (Asif et al., 2022). The characteristic SPR peak at 430 nm, observed using UV-Vis spectroscopy, is consistent with the synthesis of spherical AgNPs, as previously reported in studies involving plant extracts such as *Avena fatua* and *Eucalyptus globulus* (Qureshi et al., 2023).

FTIR analysis revealed functional groups (O-H, C=O, and C-N) from the plant extract involved in the reduction and stabilization of AgNPs. These findings are in line with the work of other researchers, such as Tassanee et al., who showed that phenolic compounds and flavonoids in plant extracts are crucial in reducing metal ions to form stable nanoparticles. The crystalline nature of the synthesized AgNPs, as confirmed by XRD, further supports the successful synthesis, with diffraction peaks corresponding to the FCC structure of silver (Ongtanasup et al., 2024). The average crystallite size of 18 nm is consistent with other studies, where nanoparticle sizes ranged from 10 to 25 nm, exhibiting optimal properties for biomedical applications (Rana et al., 2024). FESEM analysis demonstrated that the AgNPs were predominantly spherical in shape with a size range of 15–25 nm. This uniform morphology is desirable for drug delivery applications, as it enhances stability and bioavailability. Furthermore, the minimal agglomeration of the nanoparticles is advantageous for ensuring uniform distribution in biological systems. The synthesis of AgNPs using extract, therefore, offers a promising green approach to nanoparticle production with potential applications in cancer therapy (Ratan et al., 2020).

The cytotoxic effects of *S. indicus* extract, AgNPs, and cryptomeridiol were evaluated against HGT1 cells and normal hepatocytes. The ethanolic extract demonstrated moderate cytotoxicity against HGT1 cells, similar to other plant extracts such as *Cynara scolymus L* (Khedr et al., 2022) and *Cicer arietinum* (Baran et al., 2022), which also showed selective toxicity toward cancer cells while sparing normal cells. In contrast, the biosynthesized AgNPs exhibited superior anticancer activity, significantly inhibiting the growth of HGT1 cells while minimizing toxicity to normal hepatocytes. This enhanced activity of AgNPs compared to the crude extract is consistent with previous studies, where AgNPs derived from plant extracts, such as *Gymnema sylvestre* (Muthu et al., 2025), showed improved anticancer effects due to their small size and increased surface area, which allows for enhanced cellular uptake and more potent biological effects. The active fraction containing cryptomeridiol demonstrated the highest selectivity and potency in suppressing HGT1 cell proliferation, with minimal toxicity to normal hepatocytes. This finding aligns with other studies in which specific plant-derived from *Lannea barteri*, have been shown to possess selective anticancer properties (Mbaoji et al., 2020). Cryptomeridiol may act through the inhibition of key signaling pathways involved in cell proliferation and apoptosis, and further investigation into its molecular targets could help elucidate its mode of action.

The results of the CCK-8 assay revealed dose-dependent cytotoxicity for all treatments, with higher concentrations of extract, AgNPs, and cryptomeridiol resulting in reduced cell viability. This finding corroborates previous reports where plant extracts and nanoparticles demonstrated similar cytotoxicity profiles in cancer cell lines (Khorrami et al., 2018). The qPCR results provide compelling evidence that *S. indicus* extract, isolated fraction, and AgNPs induce apoptosis in HGT1 cells via a dose-dependent modulation of apoptosis-related genes. The significant upregulation of p21, a key regulator of cell cycle arrest, alongside the concurrent downregulation of CDK2, suggests that these treatments effectively halt cell cycle progression, thereby inhibiting cellular proliferation (Bhattacharya et al., 2021). The marked increase in CASP3 and CASP9 expression highlights the activation of intrinsic apoptotic pathways, as these caspases are crucial mediators of apoptosis triggered by mitochondrial signals. The upregulation of PARP1 further corroborates the pro-apoptotic effects of these treatments, as cleaved PARP is a hallmark of DNA damage and cell death. Conversely, the significant downregulation of XIAP, an anti-apoptotic gene that inhibits caspase activity, demonstrates that the treatments mitigate anti-apoptotic defenses, thereby promoting apoptosis. These findings suggest that the apoptotic effects of extract, isolated fraction, and AgNPs are mediated through the intrinsic mitochondrial pathway, making them promising candidates for therapeutic applications targeting apoptosis in cancer cells (Bhattacharya et al., 2021). Notably, the apoptosis-related protein expression analysis via Western blotting showed that treatment with *S. indicus* extract, isolated fraction, and AgNPs induced apoptosis in HGT1 cells by upregulating pro-apoptotic markers (caspase-3, caspase-9) and downregulating anti-apoptotic proteins (XIAP). These results are consistent with studies on other nanoparticle-based treatments, where AgNPs induced apoptosis in cancer cells through mitochondrial dysfunction and caspase activation (Ullah et al., 2020). The significant cleavage of PARP, a hallmark of apoptosis, further supports the apoptotic mechanism of action, as reported in similar studies where plant-derived compounds and nanoparticles triggered PARP cleavage in various cancer cell lines. The inhibition of cdk2 and the increase in p21 expression suggest that the treatments may also arrest the cell cycle at the G1 phase, preventing the proliferation of cancer cells. These findings are consistent with other reports on plant-based compounds and nanoparticles that exert cell cycle arrest in cancer cells (Abbas and Dutta, 2009). The novelty of this study lies in the use of *S. indicus* extract for the synthesis of silver nanoparticles, which exhibit both anticancer properties and selective toxicity toward cancer cells. While AgNPs have been widely studied for their therapeutic potential, the use of *S. indicus* extract for nanoparticle synthesis is a relatively unexplored approach. The green synthesis method offers an environmentally friendly and cost-effective alternative to conventional chemical synthesis methods, which often involve toxic chemicals and high-energy processes. The implications of these findings extend beyond just cancer treatment. The ability of *S. indicus* compounds to regulate apoptosis and modulate key cellular pathways underscores its broader therapeutic potential, which could include applications in other diseases where cell cycle regulation and apoptosis are critical, such as neurodegenerative diseases, autoimmune conditions, and inflammatory disorders. Additionally, the eco-friendly approach to nanoparticle synthesis using plant extracts could inspire future research into plant-based green nanotechnology, which aligns with current trends toward sustainable and less toxic alternatives in medicine. Future research directions should focus on validating these *in silico* findings through *in vitro* and *in vivo* studies to confirm the anticancer efficacy of these compounds in animal models. Further isolation and characterization of the active compounds from the chloroform fraction could lead to the identification of even more potent agents. Additionally, exploring the synergistic effects of the plant extract and silver nanoparticles in combination with conventional chemotherapy agents may yield insights into combination therapies that enhance therapeutic outcomes while reducing side effects. Long-term toxicological studies and clinical trials would be necessary to evaluate the safety and efficacy of these bioactive compounds in human patients.

## Conclusion

This study highlights the anticancer potential of *S. indicus* and its bioactive compounds. In-silico docking revealed that flavonoids and terpenoids, such as quercetin and kaempferol, strongly bind to key cancer-related proteins like p53 and Bcl-2, suggesting their role in modulating cancer pathways. Cytotoxicity assays confirmed the significant anticancer activity of the plant extract, silver nanoparticles (AgNPs), and cryptomeridiol against gastric cancer cells (HGT-1), with minimal toxicity to normal cells. These findings support the therapeutic potential of *S. indicus* in cancer treatment, warranting further *in-vivo* studies for clinical validation.

## Data Availability

The original contributions presented in the study are included in the article/Supplementary Material, further inquiries can be directed to the corresponding authors.
