# Supplementary figures and images for: Synergistic anticancer potential of biogenic nanoparticles and cryptomeridiol from Sphaeranthus indicus: targeting gastric cancer through apoptosis and cell cycle arrest
Source: Front Pharmacol. 2025 Apr 3;16:1565308. doi: 10.3389/fphar.2025.1565308 (PMC12003266; doi:10.3389/fphar.2025.1565308)

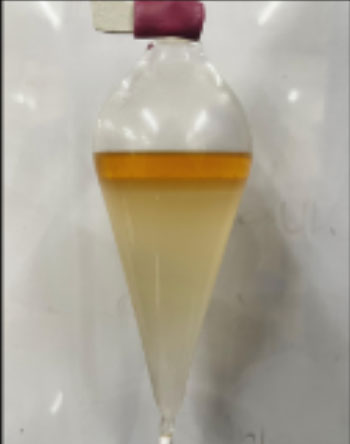

Supplement: Supplementary file 1 [file Image1.jpeg]
